# Exploring the association between short/long preceding birth intervals and child mortality: using reference birth interval children of the same mother as comparison

**DOI:** 10.1186/1471-2458-13-S3-S6

**Published:** 2013-09-17

**Authors:** Naoko Kozuki, Neff Walker

**Affiliations:** 1 Department of International Health, Bloomberg School of Public Health, Johns Hopkins University, Baltimore, MD, USA

## Abstract

**Background:**

This study used data from recent Demographic and Health Surveys (DHS) to examine the impact of short or long preceding birth intervals on neonatal and under-five mortality. In order to minimize the effect of selection issues, we examined child mortality outcomes of the same mother, comparing short or long interval births against births with what had previously been considered optimal intervals.

**Methods:**

We analyzed 47 DHS datasets from low- and middle-income countries. For each dataset, we compared neonatal and under-five mortality of short preceding interval births (<18 months, <24 months) to reference interval births (24-<60 months) of a mother, using conditional logistic regression matching on the mother. We also conducted the same matched analysis for long (≥60 months, ≥72 months) preceding interval births. These associations were then meta-analyzed. We also stratified the analyses by mothers’ completed fertility (fertility at end of reproductive period) to assess whether maternal characteristics highly correlated with completed fertility modify the association between birth interval and child mortality.

**Results:**

Children with shorter preceding intervals had increased odds of both neonatal (<24 months, OR: 1.61, 95% CI: 1.52-1.70) and under-five mortality (<24 months, OR: 1.48, 95% CI: 1.40-1.56). When the associations were stratified by the mothers’ completed fertility, the impact of short intervals was greatly reduced or eliminated for low fertility mothers. In contrast, mortality associations became stronger for children of high fertility mothers. However, when the births of high fertility mothers were limited to birth orders 2-4, the associations were comparable to those of low fertility mothers. Longer preceding birth intervals had lower odds of mortality than reference intervals (i.e. under-5 mortality for ≥60 months, OR 0.59, 95% CI: 0.52-0.67). This effect was also mediated by mothers’ completed fertility; there was a strong protective effect of longer birth intervals for the high fertility mothers but not for low fertility mothers.

**Conclusions:**

These analyses reproduced findings reported in previous literature that shorter birth intervals are associated with higher child mortality. However the negative impact of short birth intervals may only occur in high parity births. Reproductive health interventions that seek to lengthen birth intervals may have larger impact by targeting women with high parity. This finding is consistent with the concept of maternal depletion as the underlying cause of increased adverse child outcomes associated with shorter birth intervals.

## Introduction

Short and long preceding birth intervals, or time between a birth and the subsequent birth, have previously been linked to adverse child outcomes[1-5]. With short intervals, some have proposed maternal depletion as the causal mechanism; mothers may not have had enough time to recuperate physiologically from the previous birth[6,7]. Others have speculated that there may be an effect related to resource distribution within the family; if a mother has children closely spaced together, her resources in terms of food, money, time, and ability to provide for each child may be limited. For long intervals, a woman’s reproductive capacity may decline and return to the primiparous state if birth intervals are overly long.[8] There remains a possibility that the birth interval associations reported in previous literature are strongly confounded. Short intervals may be heavily correlated with poor socioeconomic status, lack of access to health care services, low education, and other factors that are associated with adverse child outcomes,[9] while long intervals may be a direct product of miscarriages and stillbirths between live births or of infertility. These maternal traits may make children vulnerable regardless of birth interval.

In our analysis, we seek to estimate the association between birth intervals and child mortality, and the extent to which this association can be explained by physiology versus environmental causes. The literature reporting birth interval associations have mainly used cross-sectional data, and have sought to remove possible confounding effects such as maternal age, parity, and socioeconomic status from the birth interval-mortality association. To better extricate possible residual confounding from the true association, we use new analytic approaches on Demographic and Health Survey (DHS) data. We compare child mortality of risky interval and reference interval births of the same mother; by examining child mortality risk with a mother as the unit, we better control for residual confounding that cannot be addressed statistically. We also restrict the analysis to births that occurred when women were ages 18-<35 to eliminate age as a potential confounder rather than relying on statistical control. Finally, we only include women who were over age 35 at the time of the survey. This allows us to derive her fertility at the end or near the end of her reproductive period (completed fertility), which we will then use as a proxy for maternal characteristics that may possibly alter the association between birth intervals and child mortality.

The overarching purpose of this paper and other papers in this supplement is to investigate the possible impact of contraceptive usage and its ensuing drop in fertility on the rates of child and maternal mortality. The findings will inform what biological links between reproductive health-related exposures and child mortality should be included in the Lives Saved Tool (*LiST*). *LiST* is a software package that estimates the impact of scaling up interventions on reducing maternal and child mortality, stillbirths, and other outcomes such as stunting and wasting in low- and middle-income countries.[10]* LiST* has been developed within Spectrum,[11] which links *LiST* to a family planning module; currently in the model, the number of pregnancies and resulting maternal and child deaths and stillbirths decrease as contraceptive use goes up. We seek to better understand the complex associations linking contraceptives, birth intervals, and their associations with child mortality, and update the *LiST* model with best available evidence.

## Methods

### Datasets

DHS are performed in many low- and middle-income countries and provide a broad range of nationally-representative information related to health. These surveys include full birth histories for women aged 15-49 living in a household. We identified a total of 49 datasets from more recent DHS surveys (Phase V: 29 Africa, 8 Asia, 5 Americas, 7 North Africa/Central Asia/Europe). We did not include surveys from Sao Tome and Principe and Ukraine due to low number of overall births and low number of high fertility births, resulting in 47 countries included in the analyses. If multiple Phase V surveys were conducted in a single country, we used the most recent survey.

### Selection of mothers and births

We first limited our analyses to children of mothers who were over 35 years of age at the time of survey. A separate analysis found that children of women with high fertility at the end of her reproductive period have higher mortality risk than those born to women with low fertility, regardless of birth order.[12] This suggests that birth interval may have differential impact on a child’s mortality, depending on characteristics associated with the mother’s completed fertility. By limiting mothers to those who were age 35 or above at the time of interview, we can obtain maternal fertility that is close to, if not exactly, the final fertility at the end of the mother’s reproductive period. We stratify the analysis by low and high fertility mothers to see if maternal characteristics correlated with completed fertility would modify the association between birth intervals and child mortality outcomes. For these women, we excluded births that occurred when they were either below 18 or over 35 years of age to eliminate age as a possible confounder.

Survival or death by 28 days (neonatal mortality) and five years of age (under-five mortality) were recorded for the included births. Preceding birth interval is available for each child in the datasets, aside from first births. When looking at under-five mortality as an outcome, we excluded any births that occurred in the five years before the mother completed the survey, as we cannot fairly assess survivorship at age five. Similarly for neonatal mortality, we did not include any births that occurred in the month prior to the mother completing the survey.

### Analysis

We examined two different cut-offs for short intervals (<18 months and <24 months) and two different cut-offs for long intervals (≥60 months and ≥72 months). 24-<60 month was used as the reference interval for all analyses, based on previous literature reporting this interval as optimal. We conducted conditional logistic regression for each of the aforementioned short or long intervals as the exposed group, matched on the mother. Then, the same analyses were conducted stratified by maternal completed fertility. Mothers who had four or fewer live births were categorized as low completed fertility and mothers who had five or more live births were categorized as high completed fertility, given the mothers were age 35 or older at the time of the survey. (All mentions of fertility hereafter refer to the mothers’ completed fertility.) In order to infer if short or long birth intervals make an impact on all births or only following a cumulative effect after numerous births, we re-ran the same analysis for high fertility mothers, but limiting the children included in the analysis to birth orders 2-4.

For each of the above analyses, the country-level associations were meta-analyzed using the metan command in Stata. We also meta-analyzed the country-level associations by DHS region (Sub-Saharan Africa, Americas, Asia, North Africa/Central Asia/Europe). We used random effects models for the meta-analysis to control for heterogeneity across countries. Stata Version 12 was used for the analyses.

## Results

We present the results in two sections. First we show the neonatal and under-5 mortality odds ratios for short preceding birth intervals and then for long preceding birth intervals. We then run the same analyses stratified by mothers’ completed fertility. For both sets of analyses, we also present the results by region. The sample sizes and odds ratios by country are available in Additional File 1 Supplemental Tables 1a-b for under-5 mortality and Supplemental Tables 2a-b for neonatal mortality.

### Short interval

**Figure 1:**
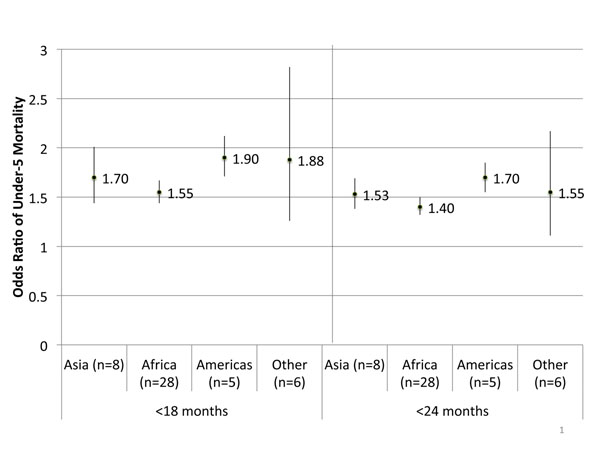
**Meta-analyzed odds ratios between short birth intervals and under-5 mortality, by region.** Reference birth interval: 24-<60 months

We found that children who are born after short birth intervals have increased odds of both neonatal and under-5 mortality compared to their siblings born after a regular birth interval. Having a <18 month interval had 82% increased odds (95% CI: 1.55-1.79) of neonatal mortality and 66% increased odds (95% CI: 1.55, 1.79) of under-five mortality, compared to those born after 24-<60 month intervals. The <24 month interval had a slightly attenuated effect size of 61% increased odds (95% CI: 1.52, 1.70) of neonatal mortality and 48% increased odds (95% CI: 1.40, 1.56) of under-five mortality. (See Table 1 for odds ratios for short birth intervals). This adverse association of short birth intervals remained when the mortality risks were stratified by region. In general, Africa had the smallest effect size and the Americas the largest. (See Figure 1 for under-five mortality associations. Neonatal mortality associations not pictured.)

**Table 1:** Meta-analyzed odds ratios for risky birth intervals, with neonatal and under-5 mortality as outcomes

Region (number of countries)	Short Interval		Reference Interval	Long Interval	
	
	< 18 months OR (95% CI)	< 24 months OR (95% CI)	24-<60 months	≥60 months OR (95% CI)	≥72 months OR (95% CI)
**Outcome: Neonatal Mortality**					

Africa (n=28)	1.79 (1.54, 2.09)	1.66 (1.55, 1.78)	Ref	0.81 (0.59, 1.11)	0.75 (0.54, 1.05)
Asia (n=8)	1.74 (1.62, 1.87)	1.55 (1.47, 1.63)	Ref	0.73 (0.59, 0.92)	0.83 (0.62, 1.11)
Americas n = 5)	2.15 (1.80, 2.57)	1.82 (1.57, 2.12)	Ref	0.77 (0.47, 1.29)	0.89 (0.42, 1.86)
Other (n = 6)	1.76 (1.01, 3.05)	1.32 (0.77, 2.27)	Ref	1.81 (0.77, 4.26)	1.46 (0.63, 3.42)*
All (n=47)	1.82 (1.70, 1.95)	1.61 (1.52, 1.70)	Ref	0.80 (0.67, 0.95)	0.85 (0.69, 1.04)

**Outcome: Under-5 Mortality**					

Africa (n=28)	1.55 (1.44, 1.67)	1.40 (1.32, 1.50)	Ref	0.55 (0.47, 0.65)	0.55 (0.45, 0.68)
Asia (n=8)	1.70 (1.44, 2.01)	1.53 (1.38, 1.69)	Ref	0.60 (0.45, 0.79)	0.54 (0.41, 0.73)
Americas n = 5)	1.90 (1.71, 2.12)	1.70 (1.55, 1.85)	Ref	0.69 (0.48, 0.98)	0.73 (0.54, 0.99)
Other (n = 6)	1.88 (1.26, 2.82)	1.55 (1.11, 2.17)	Ref	0.89 (0.62, 1.28)	0.73 (0.27, 1.94)
All (n=47)	1.66 (1.55, 1.79)	1.48 (1.40, 1.56)	Ref	0.59 (0.52, 0.67)	0.58 (0.49, 0.67)

Reference birth interval: 24-<60 months

### Long interval

**Figure 2:**
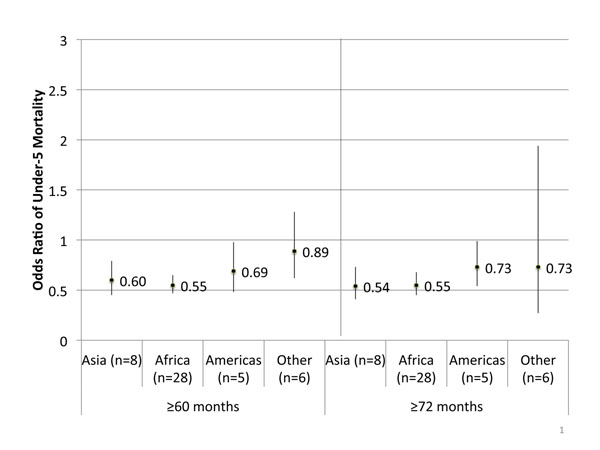
**Meta-analyzed odds ratios between long birth intervals and under-5 mortality, by region.** Reference birth interval: 24-<60 months

Contrary to other studies, we found that children who are born after long birth intervals have lower odds of neonatal and under-five mortality than their siblings born after a reference birth interval. Having ≥60 month birth interval had 20% decreased odds (95% CI: 0.67-0.95) of neonatal mortality and 41% decreased odds (95% CI: 0.52-0.67) of under-five mortality compared to their siblings born after a 24-<60 month interval. The odds of mortality remained similar when examining birth interval ≥72 months. (See Table 1) When analyzed by region, only Asia for the ≥60 month cut-off had statistically significant protective odds for neonatal mortality. For under-five mortality the lower odds of death remained significant for Africa, Asia and Latin America for both ≥60 and ≥72 month intervals. There was no statistically significant association for the North Africa/Central Asia/Europe region. (See Figure 2 for under-five mortality associations, neonatal mortality associations not pictured.)

### Stratification by maternal completed fertility

In a separate study in this supplement, we saw large differences in mortality risk between children of high completed fertility (five or more live births) and low completed fertility (four or fewer live births) mothers, even after controlling for confounders [12]. The differences in characteristics between these two categories of mothers are reflected in Supplemental Table 2 of the aforementioned study [12], where we compare socioeconomic backgrounds (maternal education, wealth quintile, proportion living in rural areas) of high fertility and low fertility mothers in each country. We see a consistent trend across countries that low completed fertility women are better off socioeconomically, and this trend remained when statistics were averaged across all countries. All of these characteristics are generally correlated with better health and lower mortality. Noting these background differences, we stratified the birth interval associations by maternal completed fertility to see if short or long birth intervals have a differential impact on child mortality.

### Short interval

**Figure 3:**
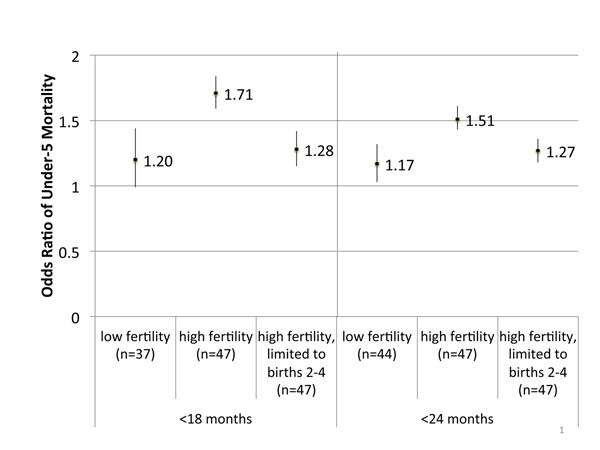
**Meta-analyzed odds ratios between short birth intervals under-5 mortality, stratified by mothers’ completed fertility.** Reference birth interval: 24-<60 months, Completed fertility: woman’s total fertility at the end of her reproductive period

Among low completed fertility women, there were no statistically significant associations with neonatal mortality with short birth intervals. For under-five mortality, there was no statistically significant association for the <18 months (OR 1.20, 95% CI: 0.99-1.44) but a statistically significant association for the <24 months period (OR 1.17, 95% CI 1.03-1.32). For the high completed fertility women, the odds ratios for neonatal and under-five mortality were statistically significant for both the <18 and <24 month intervals. (See Figure 3 for associations with under-five mortality, Table 2 for all associations.)

**Table 2:** Meta-analyzed odds ratios for short birth intervals with neonatal and under-5 mortality as outcomes, stratified by mother's completed fertility

Region (number of countries)	Interval < 18 months		Interval < 24 months	
	
	Low Fertility OR (95% CI)	High fertility OR (95% CI)	Low Fertility OR (95% CI)	High fertility OR (95% CI)
**Outcome: Neonatal Mortality**				

Africa (n=28)	0.85 (0.53, 1.34)	1.80 (1.66, 1.94)	0.87 (0.65, 1.17)	1.59 (1.49, 1.69)
Asia (n=8)	1.20 (0.91, 1.57)	1.89 (1.61, 2.22)	1.23 (0.94, 1.63)	1.71 (1.59, 1.85)
Americas n = 5)	1.56 (0.84, 2.89)	2.24 (1.86, 2.70)	1.15 (0.77, 1.71)	1.97 (1.69, 2.28)
Other (n = 6)	0.86 (0.31, 2.35)	2.59 (1.71, 3.94)	0.71 (0.35, 1.43)	2.01 (1.28, 3.18)
All (n=47)	1.07 (0.84, 1.37)	1.91 (1.77, 2.05)	1.06 (0.90, 1.25)	1.67 (1.58, 1.77)

**Outcome: Under-5 Mortality**				

Africa (n=28)	0.86 (0.64, 1.14)	1.59 (1.47, 1.71)	1.06 (0.89, 1.27)	1.42 (1.33, 1.52)
Asia (n=8)	1.28 (1.07, 1.55)	1.77 (1.50, 2.09)	1.05 (0.82, 1.35)	1.61 (1.46, 1.76)
Americas n = 5)	1.72 (1.18, 2.52)	1.92 (1.72, 2.14)	1.48 (1.08, 2.01)	1.73 (1.59, 1.89)
Other (n = 6)	1.74 (0.99, 3.05)	2.12 (1.46, 3.08)	1.39 (0.89, 2.16)	1.74 (1.27, 2.37)
All (n=47)	1.20 (0.99, 1.44)	1.71 (1.59, 1.84)	1.17 (1.03, 1.32)	1.51 (1.43, 1.61)

Reference birth interval: 24-<60 months

Completed fertility: woman’s total fertility at the end of her reproductive period

We found similar effects when the results above were stratified by region. Among low completed fertility women, associations between short intervals (both <18 and <24 month cut-offs) and neonatal mortality were not statistically significant in all regions. For under-5 mortality, there were mixed findings depending on the cut-off and region. In contrast, for high fertility women, the increased odds for neonatal and under-five mortality were statistically significant in all regions for both the <18 and <24 month intervals. (Table 2.)

The differential impact of short birth intervals on mothers with different fertility histories could be due to background differences between the two groups. However, it could also be due to short birth intervals having a cumulative negative effect, making later births more sensitive to the deleterious effect of short intervals. To further explore this issue, we re-ran the analyses, limiting the analyzed births of high fertility mothers to birth orders 2-4. As shown in Figure 3 (for under-five mortality) and Table 3, there were still significant effects of both <18 and <24 month intervals on both neonatal and under-five mortality, however these odds ratios were much lower than the odds ratios that included all births of high-fertility mothers.

**Table 3:** Meta-analyzed odds ratios for short birth intervals with neonatal and under-5 mortality as outcomes, comparing all children versus birth order 2-4 children of high completed fertility mothers

	Interval < 18 months		Interval < 24 months	
**Region (number of countries)**	**High fertility OR (95% CI)**	**High fertility, limited to birth order 2-4 OR (95% CI)**	**High fertility OR (95% CI)**	**High fertility, limited to birth order 2-4 OR (95% CI)**

**Outcome: Neonatal Mortality**				

Africa (n=28)	1.80 (1.66, 1.94)	1.16 (1.02, 1.33)	1.59 (1.49, 1.69)	1.21 (1.11, 1.33)
Asia (n=8)	1.89 (1.61, 2.22)	1.52 (1.17, 1.97)	1.71 (1.59, 1.85)	1.46 (1.27, 1.67)
Americas n = 5)	2.24 (1.86, 2.70)	1.78 (1.35, 2.33)	1.97 (1.69, 2.28)	1.72 (1.40, 2.10)
Other (n = 6)	2.59 (1.71, 3.94)	2.18 (1.05, 4.54)	2.01 (1.28, 3.18)	1.78 (0.92, 3.45)
All (n=47)	1.91 (1.77, 2.05)	1.36 (1.19, 1.55)	1.67 (1.58, 1.77)	1.34 (1.23, 1.46)

**Outcome: Under-5 Mortality**				

Africa (n=28)	1.59 (1.47, 1.71)	1.15 (1.03, 1.28)	1.42 (1.33, 1.52)	1.18 (1.10, 1.27)
Asia (n=8)	1.77 (1.50, 2.09)	1.35 (1.10, 1.65)	1.61 (1.46, 1.76)	1.33 (1.15, 1.56)
Americas n = 5)	1.92 (1.72, 2.14)	1.63 (1.37, 1.94)	1.73 (1.59, 1.89)	1.55 (1.37, 1.75)
Other (n = 6)	2.12 (1.46, 3.08)	1.48 (0.82, 2.66)	1.74 (1.27, 2.37)	1.29 (0.84, 2.00)
All (n=47)	1.71 (1.59, 1.84)	1.28 (1.15, 1.42)	1.51 (1.43, 1.61)	1.27 (1.18, 1.36)

Reference birth interval: 24-<60 months

Completed fertility: woman’s total fertility at the end of her reproductive period

### Long interval

**Figure 4:**
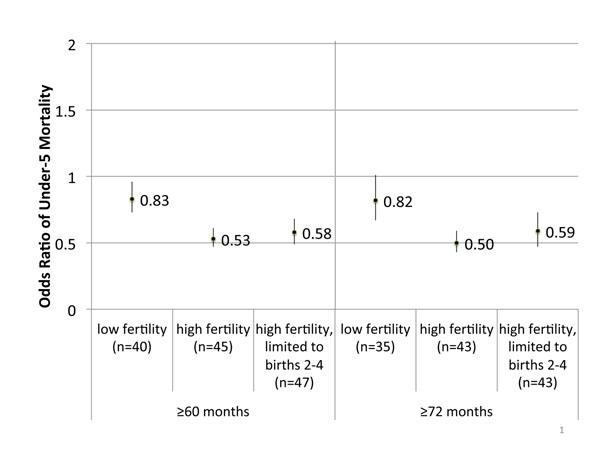
**Meta-analyzed odds ratios between long birth intervals under-5 mortality, stratified by mothers’ completed fertility.** Reference birth interval: 24-<60 months, Completed fertility: woman’s total fertility at the end of her reproductive period

We conducted the same analysis for long birth intervals to examine if the protective association between long birth intervals and mortality differs by fertility category of the mother. The protective association of long birth intervals became weaker in the low fertility mothers while it became stronger in the high fertility mothers for both under-5 and neonatal mortality. (See Figure 4 for under-five mortality, Table 4 for all associations.) For high fertility women, the odds ratios for neonatal and under-five mortality were statistically significant for both ≥60 and ≥72 month cut-offs. For low fertility mothers, there were weak or no statistically significant protective associations. Note that several countries did not contribute odds ratios, as they had a very small sample of births and/or deaths in the longer birth interval categories when stratified by fertility. When the high fertility mothers’ births were limited to birth order 2-4, there were no statistically significant changes to the associations, unlike the shift we saw for short birth intervals (See Figure 4 for under-five mortality, neonatal mortality not pictured). This held true for the combined associations and the region-stratified associations (Table 5).

**Table 4:** Meta-analyzed odds ratios for long birth intervals with neonatal and under-5 mortality as outcomes, stratified by mothers' completed fertility

	Interval ≥60 months		Interval ≥72 months	
**Region (number of countries)**	**Low Fertility OR (95% CI)**	**High fertility OR (95% CI)**	**Low Fertility OR (95% CI)**	**High fertility OR (95% CI)**

**Outcome: Neonatal Mortality**				

Africa (n=28)	0.96 (0.65, 1.40)	0.70 (0.54, 0.92)	1.01 (0.62, 1.64)	0.78 (0.58, 1.05)
Asia (n=8)	1.15 (0.75, 1.77)	0.60 (0.44, 0.81)	0.91 (0.61, 1.37)	0.56 (0.40, 0.77)
Americas (n = 5)	1.26 (0.76, 2.09)	0.67 (0.41, 1.10)*	1.26 (0.67, 2.38)	0.79 (0.35, 1.79)*
Other (n = 6)	3.37 (0.94, 12.09)	1.13 (0.39, 3.30)	3.49 (0.67, 18.05)**	1.15 (0.33, 4.06)***
All (n=47)	1.14 (0.89, 1.45)	0.69 (0.57, 0.84)	1.04 (0.79, 1.36)	0.75 (0.60, 0.93)

**Outcome: Under-5 Mortality**				

Africa (n=28)	0.84 (0.67, 1.05)	0.52 (0.44, 0.61)	0.85 (0.63, 1.14)	0.51 (0.42, 0.62)
Asia (n=8)	0.77 (0.61, 0.98)	0.49 (0.36, 0.68)	0.75 (0.53, 1.05)	0.38 (0.29, 0.49)
Americas (n = 5)	0.94 (0.57, 1.53)	0.59 (0.44, 0.78)	0.83 (0.46, 1.49)	0.62 (0.42, 0.91)*
Other (n = 6)	1.07 (0.57, 1.98)	0.78 (0.49, 1.25)	0.87 (0.24, 3.22)**	0.71 (0.33, 1.53)***
All (n=47)	0.83 (0.73, 0.96)	0.53 (0.47, 0.61)	0.82 (0.67, 1.01)	0.50 (0.43, 0.59)

*n=5, **n=3, ***n=2

Reference birth interval: 24-<60 months

Completed fertility: woman’s total fertility at the end of her reproductive period

**Table 5:** Meta-analyzed odds ratios for long birth intervals with neonatal and under-5 mortality as outcomes, comparing all children versus birth order 2-4 children of high fertility mothers

	Interval ≥60 months		Interval ≥72 months	
**Region (number of countries)**	**High fertility OR (95% CI)**	**High fertility, limited to birth order 2-4 OR (95% CI)**	**High fertility OR (95% CI)**	**High fertility, limited to birth order 2-4 OR (95% CI)**

**Outcome: Neonatal Mortality**				

Africa (n=28)	0.70 (0.54, 0.92)	0.82 (0.62, 1.08)	0.78 (0.58, 1.05)	0.84 (0.60, 1.18)
Asia (n=8)	0.60 (0.44, 0.81)	0.72 (0.38, 1.36)	0.56 (0.40, 0.77)	0.57 (0.32, 1.02)
Americas n = 5)	0.67 (0.41, 1.10)*	0.58 (0.31, 1.09)*	0.79 (0.35, 1.79)*	0.85 (0.32, 2.24)*
Other (n = 6)	1.13 (0.39, 3.30)	1.17 (0.38, 3.64)***	1.15 (0.33, 4.06)***	1.54 (0.31, 7.60)****
All (n=47)	0.69 (0.57, 0.84)	0.76 (0.61, 0.94)	0.75 (0.60, 0.93)	0.76 (0.58, 0.98)

**Outcome: Under-5 Mortality**				

Africa (n=28)	0.52 (0.44, 0.61)	0.58 (0.48, 0.70)	0.51 (0.42, 0.62)	0.58 (0.45, 0.75)
Asia (n=8)	0.49 (0.36, 0.68)	0.52 (0.36, 0.74)	0.38 (0.29, 0.49)	0.40 (0.27, 0.59)
Americas n = 5)	0.59 (0.44, 0.78)	0.55 (0.35, 0.85)	0.62 (0.42, 0.91)	0.82 (0.47, 1.43)
Other (n = 6)	0.78 (0.49, 1.25)	1.26 (0.59, 2.69)**	0.71 (0.33, 1.53)	4.18 (0.93, 18.84)****
All (n=47)	0.53 (0.47, 0.61)	0.58 (0.49, 0.68)	0.50 (0.43, 0.59)	0.59 (0.47, 0.73)

*n=5, **n=3, ***n=2, ****n=1

Reference birth interval: 24-<60 months

Completed fertility: woman’s total fertility at the end of her reproductive period

When stratified by region, the statistically significantly protective association among children of high fertility mothers remained in all regions but the North Africa/Central Asia/Europe region. On the other hand, the associations among children of low fertility mothers were not statistically significant across all regions, except Asia for the ≥60 month cut-off (Table 4). As there are higher proportions of high fertility mothers in Africa and lower proportions of high fertility mothers in North Africa/Central Asia/Europe (See Additional file 1 Supplemental Table 3), the regional differences in associations for both short intervals (Table 2) and long intervals (Table 4) may be explained by the population-level composition of high versus low fertility mothers and the differences in birth interval-mortality associations by fertility level.

A separate paper in this supplement demonstrated that parity alone does not have a large physiological impact, if at all, on child mortality [12], which removed the need to control for birth order. However, for descriptive purposes, we summarized the average birth orders of the included short, reference, and long birth intervals by country. We found no systematic tendencies for short, reference, or long birth intervals to occur in particular birth order (Additional file 1 Supplemental Table 4). We also saw no systematic trends of certain birth intervals occurring on certain years, relative to the time of the survey (Additional file 1 Supplemental Table 5).

## Conclusions

Overall, our analyses found that short preceding birth intervals (<18 months, <24 months) have higher odds of neonatal and under-five mortality, a finding that is consistent with most previous findings.[2,13] However, in contrast to previous findings, our analyses found that long preceding birth intervals (≥60 months, ≥72 months) have lower odds of mortality. By making mortality comparisons among births of the same mother, we believe we have controlled for most socioeconomic and other background factors that could have confounded previous cross-sectional analyses on the impact of birth intervals on child mortality. We do not expect large or systematic changes in the mother’s socioeconomic or other background characteristics throughout her reproductive life to affect our findings, and we believe that any maternal age confounding is sufficiently limited by examining only births that occurred when the mothers were age 18-<35.

While the overall effects of short intervals on neonatal and under-five mortality were consistent with findings from previous studies, our analyses also found major differences in odds ratios related to fertility history of mothers. When the associations were stratified by maternal completed fertility, the association between short birth intervals and child mortality largely disappeared for mothers with low completed fertility (had four or fewer live births at the end of their reproductive period). Furthermore, odds ratios tend to report larger magnitude associations than relative risks, a more easily interpretable measure of association.[14] While we do not expect the effect sizes to differ widely because of the rarity of the mortality outcomes, even the statistically significant associations that remained may be minimal or non-existent if we had examined relative risks instead.

In contrast, children of mothers with high completed fertility (had five or more live births by the end of their reproductive period) had strong associations between short intervals and mortality. However, when we limited the births included in the analysis for high fertility mothers to birth order 2-4, the magnitude of the associations were attenuated while remaining statistically significant. This suggests that there may be complex interactions between short birth intervals, differences across low- and high-fertility women, and parity.

These findings seem to be consistent with the theory that maternal depletion is responsible for the higher mortality risks associated with short birth spacing.[6,7] Low fertility mothers may have better nutritional status and access to care, hence short birth intervals may not deplete the mother’s nutritional resources to a level that results in increased risk of mortality for the child. For high fertility mothers, the women may be starting out with worse nutritional status and access to care. A short interval birth can deplete the mother’s resources to a level that results in increased risk of mortality for the child, but this effect may be even greater for later births; later births may be more vulnerable to short birth intervals as a result of mothers’ repeated exposure to nutritional depletion.

The maternal depletion explanation is also consistent with our findings for longer birth intervals. The protective effect against mortality of long intervals was primarily seen among the high fertility women. However, among these women, the protective effective was similar comparing all births to just their earlier births; considering our previous theory regarding the cumulative adverse effect of short intervals, we expected the protective effect to also be smaller among the earlier births and greater for later births.

Winkvist et al. [9] classified women into five different patterns of energy balance during a reproductive cycle in order to better define maternal depletion syndrome. Those who maintain equilibrium or have increased energy during pregnancy make up the first two categories, and a group who has decreased energy but has a long enough potential repletion phase (PRP) to regain equilibrium belongs in a third category. The fourth category of women has both negative energy intake and a short PRP, and Winkvist et al. defines this particular pattern where the short PRP is the driver of depletion as “maternal depletion syndrome.” Finally the fifth category comprises of women who are so undernourished that regardless of how long the repletion intervals are, they remain negative in energy balance. From the associations we saw in our analyses, we suspect that most of the high fertility women included in our study belong to the third and fourth categories. However, the much higher fertility women may have characteristics that place them in the non-repleteable fifth category.

In contrast to our findings, previous studies have reported increased risk of neonatal and infant mortality associated with long birth intervals, even using cut-offs higher than the ones we used here. However, the previously reported adverse impact of long birth intervals may be largely affected by residual confounding. For instance, Rutstein [1] uses a cut-off of 96 months for birth-to-conception interval, or approximately 105 months birth-to-birth interval, a 9-year gap between births. A large proportion of mothers experiencing such long intervals may be those who have experienced reproductive difficulties rather than those who consciously used family planning to delay the birth. Women who belong to the former category may be malnourished, be experiencing infections, or have other negative characteristics, and it could be those characteristics, rather than the long birth intervals, that drive their children’s heightened mortality risk.

Our findings imply that lengthening birth intervals may only be physiologically beneficial to higher birth orders. Reproductive health programs may benefit more from targeting interventions to women with high parity. In contexts like India where there is a high prevalence of sterilization after a woman achieves her ideal of two or three children [15], lengthening birth intervals may not be a high priority. However, Winkvist’s interpretation of the maternal depletion syndrome also implies that for maximum impact, reproductive health and nutritional interventions would need to be tailored to the distinct types of mothers to improve their birth outcomes, as lengthening birth intervals may not be enough.

Our study presents the impact of birth intervals on child mortality, using an innovative method of controlling for residual confounding by comparing short and long interval births to regular interval births of the same mother. Furthermore, other literature has not addressed or identified how maternal background characteristics modify the association between birth interval and mortality. This leads us to reevaluate previous findings on this topic, and reexamine the actual impact of short or long birth intervals on a child’s mortality risk.

For *LiST*, these analyses suggest that there is a causal linkage between short birth spacing and mortality in children. Other papers in this supplement will try to further identify this mechanism and help quantify the effects to be included in the model.

## Competing interests

The authors declare that they have no competing interests.

## Authors’ contributions

NK and NW both worked on all aspects of the work for this manuscript.

## Supplementary Material

Additional file 1 Sample sizes and odds ratios for all outputs. Click here for file

## Declarations

The publication costs for this supplement were funded by a grant from the Bill & Melinda Gates Foundation to the US Fund for UNICEF (grant 43386 to "Promote evidence-based decision making in designing maternal, neonatal, and child health interventions in low- and middle-income countries”). The Supplement Editor is the principal investigator and lead in the development of the Lives Saved Tool (LiST), supported by grant 43386. He declares that he has no competing interests.

This article has been published as part of *BMC Public Health* Volume 13 Supplement 3, 2013: The Lives Saved Tool in 2013: new capabilities and applications. The full contents of the supplement are available online at http://www.biomedcentral.com/bmcpublichealth/supplements/13/S3.
