# Supplementary material for: Exploring the association between short/long preceding birth intervals and child mortality: using reference birth interval children of the same mother as comparison
Source: BMC Public Health. 2013 Sep 17;13(Suppl 3):S6. doi: 10.1186/1471-2458-13-S3-S6 (PMC3847658; doi:10.1186/1471-2458-13-S3-S6)
Supplement: Additional file 1 — Sample sizes and odds ratios for all outputs. [file 1471-2458-13-S3-S6-S1.pdf]

Kozuki N and Walker N. Exploring the association between short/long preceding birth intervals and child mortality: using reference birth interval children of the same mother as comparison.

Supplemental Table 1a: Sample sizes and odds ratios for short birth intervals, with under-5 mortality as outcome (reference birth interval: 24-<60 months)

	<18 months				<24 months			
country	number of mothers contributing to analysis	OR	95% CI: Lower	95% CI: Upper	number of mothers contributing to analysis	OR	95% CI: Lower	95% CI: Upper
**Africa**								
Benin	848	1.45	1.19	1.77	2017	1.39	1.23	1.59
Burkina Faso	987	1.15	0.95	1.39	2122	1.2	1.06	1.36
Cameroon	709	1.34	1.02	1.75	1406	1.22	1.02	1.45
Chad	525	1.7	1.33	2.17	1133	1.45	1.22	1.71
Congo-Brazzaville	278	1.35	0.82	2.21	600	1.38	1.01	1.87
DRC	675	1.74	1.34	2.56	1345	1.49	1.27	1.74
Ethiopia	1195	1.83	1.54	2.17	2000	1.56	1.37	1.79
Ghana	237	2.06	1.34	3.18	536	1.65	1.23	2.23
Guinea	562	0.84	0.66	1.05	1320	0.79	0.69	0.91
Guyana	369	2.75	1.39	5.44	595	2.04	1.14	3.65
Kenya	615	1.84	1.26	2.67	1091	1.57	1.18	2.09
Lesotho	191	1.49	0.85	2.59	422	1.27	0.85	1.9
Liberia	563	1.19	0.91	1.56	1049	1.36	1.14	1.62
Madagascar	1341	1.66	1.39	2.00	2380	1.52	1.31	1.75
Malawi	3006	1.76	1.44	2.14	1368	1.64	1.44	1.87
Mali	1389	1.46	1.19	1.78	2745	1.34	1.2	1.50
Mozambique	734	1.54	1.25	1.88	1653	1.27	1.10	1.46
Namibia	387	2.45	1.58	3.80	732	1.87	1.39	2.51
Niger	999	1.79	1.46	2.18	1784	1.41	1.23	1.61
Nigeria	2777	1.48	1.34	1.63	5408	1.31	1.22	1.41
Rwanda	955	1.42	1.17	1.71	1767	1.39	1.22	1.59
Senegal	1045	1.60	1.31	1.97	2058	1.45	1.26	1.66
Sierra Leone	419	1.58	1.18	2.12	880	1.22	1.03	1.45
Swaziland	273	1.64	1.02	2.65	554	1.59	1.13	2.24
Tanzania	631	1.71	1.32	2.22	1379	1.66	1.39	1.97
Uganda	784	1.55	1.27	1.90	1481	1.52	1.31	1.77
Zambia	428	1.93	1.44	2.58	857	1.66	1.35	2.06
Zimbabwe	309	1.47	0.77	2.82	331	1.6	0.98	2.62
**Asia**								
Bangladesh	651	1.25	0.95	1.65	1382	1.36	1.13	1.65
Cambodia	1219	1.53	1.22	1.94	2305	1.44	1.20	1.71
India	7673	2.05	1.88	2.23	14132	1.73	1.62	1.84
Indonesia	2130	1.18	0.95	1.47	3711	1.21	1.00	1.46
Maldives	928	1.66	1.24	2.23	1425	1.45	1.15	1.84

Nepal	807	1.92	1.46	2.53	1570	1.66	1.36	2.04
Pakistan	1794	2.14	1.76	2.61	2568	1.80	1.53	2.13
Philippines	1056	2.10	1.59	2.77	1750	1.54	1.23	1.93
**Americas**								
Bolivia	1348	2.13	1.76	2.57	2310	1.86	1.61	2.15
Colombia	2461	1.98	1.51	2.61	4099	1.55	1.25	1.94
Dominican Republic	2222	1.81	1.32	2.50	3368	1.57	1.21	2.04
Haiti	839	1.60	1.30	1.98	1496	1.54	1.30	1.82
Honduras	1652	1.97	1.58	2.47	2756	1.83	1.52	2.19
**Other**								
Albania	318	2.05	1.13	3.70	681	1.62	1.02	2.57
Armenia	382	1.35	0.75	2.43	588	1.20	0.78	1.86
Azerbaijan	649	1.64	1.05	2.56	973	1.58	1.07	2.34
Egypt	1935	3.43	2.81	4.20	3010	2.55	2.17	3.01
Jordan	2330	1.88	1.19	2.97	3228	1.46	0.96	2.20
Moldova	150	1.13	0.54	2.36	293	1.01	0.60	1.68

Supplemental Table 1b: Sample sizes and odds ratios relative risk for long birth intervals, with under-5 mortality as outcome (reference birth interval: 24-<60 months)

	≥60 months				≥72 months			
country	Number of mothers contributing to analysis	OR	95% CI: Lower	95% CI: Upper	Number of mothers contributing to analysis	OR	95% CI: Lower	95% CI: Upper
**Africa**								
Benin	722	0.68	0.49	0.93	351	0.75	0.46	1.21
Burkina Faso	683	0.48	0.34	0.69	328	0.37	0.22	0.63
Cameroon	445	0.38	0.22	0.65	328	0.46	0.22	0.95
Chad	262	0.26	0.13	0.52	139	0.22	0.08	0.60
Congo-Brazzaville	465	0.94	0.58	1.53	281	0.82	0.42	1.62
DRC	511	0.59	0.36	0.97	286	0.88	0.43	1.80
Ethiopia	783	0.43	0.29	0.63	421	0.29	0.16	0.53
Ghana	372	0.60	0.33	1.08	220	0.69	0.31	1.53
Guinea	557	0.26	0.17	0.41	309	0.31	0.17	0.56
Guyana	327	0.93	0.40	2.16	206	0.50	0.17	1.51
Kenya	472	0.89	0.50	1.58	257	1.42	0.62	3.25
Lesotho	450	1.50	0.80	2.80	258	1.55	0.71	3.43
Liberia	597	0.70	0.48	1.00	374	0.56	0.33	0.95
Madagascar	932	0.50	0.32	0.78	545	0.87	0.50	1.52
Malawi	246	0.5	0.37	0.67	742	0.54	0.36	0.79
Mali	676	0.29	0.19	0.44	360	0.25	0.14	0.44
Mozambique	684	0.45	0.28	0.73	394	0.54	0.28	1.03
Namibia	656	1.80	1.05	3.10	372	1.87	0.92	3.82
Niger	524	0.60	0.41	0.89	264	0.71	0.39	1.30
Nigeria	1812	0.40	0.33	0.50	1047	0.42	0.31	0.56
Rwanda	379	0.73	0.47	1.12	174	0.44	0.22	0.90
Senegal	737	0.61	0.37	1.02	418	0.80	0.39	1.65
Sierra Leone	497	0.36	0.23	0.56	312	0.25	0.14	0.44
Swaziland	342	0.50	0.23	1.10	205	0.42	0.14	1.26
Tanzania	625	0.68	0.4	1.16	332	0.48	0.23	0.99
Uganda	422	0.41	0.26	0.64	223	0.42	0.23	0.79
Zambia	366	0.30	0.18	0.52	191	0.23	0.11	0.48
Zimbabwe	605	1.00	0.56	1.76	331	1.75	0.90	3.42
**Asia**								
Bangladesh	980	0.56	0.40	0.80	585	0.44	0.27	0.71
Cambodia	910	0.79	0.53	1.17	486	0.87	0.53	1.43
India	4054	0.37	0.30	0.46	2044	0.35	0.26	0.46
Indonesia	2873	0.63	0.44	0.90	1825	0.56	0.34	0.93
Maldives	692	0.55	0.30	0.99	400	0.54	0.25	1.17

Nepal	415	0.36	0.17	0.74	184	0.41	0.14	1.23
Pakistan	534	0.88	0.51	1.52	266	0.79	0.36	1.74
Philippines	548	1.04	0.54	1.99	281	0.86	0.34	2.16
**Americas**								
Bolivia	929	0.50	0.33	0.77	561	0.49	0.27	0.89
Colombia	2710	1.06	0.72	1.58	1711	0.96	0.57	1.59
Dominican Republic	1249	0.85	0.50	1.44	739	0.99	0.49	2.01
Haiti	350	0.37	0.16	0.83	196	0.46	0.15	1.38
Honduras	937	0.69	0.41	1.18	509	0.68	0.35	1.33
**Other**								
Albania	347	0.73	0.23	2.33	148	0.05	0.01	0.39
Armenia	207	1.45	0.52	4.03	123	2.25	0.50	10.14
Azerbaijan	143	1.34	0.40	4.55	63	0.89	0.11	6.99
Egypt	1239	0.73	0.44	1.21	628	0.67	0.31	1.44
Jordan	522	0.67	0.20	2.27	228	1.08	0.16	7.20
Moldova	234	1.55	0.45	5.30	131	1.98	0.27	14.35

Kozuki N and Walker N. Exploring the association between short/long preceding birth intervals and child mortality: using reference birth interval children of the same mother as comparison.

Supplemental Table 2a: Sample sizes and odds ratios for short birth intervals, with neonatal mortality as outcome (reference birth interval: 24-<60 months)

	<18 months				<24 months			
country	Number of mothers contributing to analysis	Adj. OR	95% CI: Lower	95% CI: Upper	Number of mothers contributing to analysis	Adj. OR	95% CI: Lower	95% CI: Upper
**Africa**								
Benin	885	1.45	1.05	2.01	2089	1.43	1.14	1.80
Burkina Faso	1013	1.83	1.29	2.60	2175	1.65	1.31	2.07
Cameroon	728	1.93	1.26	2.95	1437	1.46	1.05	2.03
Chad	548	2.06	1.38	3.08	1165	1.58	1.18	2.12
Congo-Brazzaville	293	1.43	0.83	2.47	623	1.34	0.95	1.88
DRC	714	0.99	0.40	2.49	1402	1.02	0.61	1.71
Ethiopia	1233	1.64	1.19	2.25	2066	1.42	1.10	1.84
Ghana	247	2.49	1.38	4.50	558	2.06	1.35	3.14
Guinea	585	1.23	0.87	1.73	1364	1.12	0.90	1.41
Guyana	376	2.36	0.84	6.64	610	2.01	0.86	4.70
Kenya	641	1.91	1.15	3.17	1127	1.43	0.92	2.25
Lesotho	195	1.25	0.62	2.52	433	1.53	0.95	2.47
Liberia	590	1.89	1.19	2.99	1085	1.98	1.38	2.84
Madagascar	1392	1.98	1.36	2.86	2462	1.61	1.20	2.16
Malawi	1519	1.58	1.12	2.22	3106	1.64	1.27	2.13
Mali	1452	1.86	1.46	2.38	2828	1.72	1.43	2.07
Mozambique	751	1.65	1.11	2.44	1701	1.72	1.29	2.29
Namibia	401	3.78	1.75	8.18	752	2.82	1.66	4.79
Niger	1027	1.90	1.36	2.65	1838	1.55	1.20	2.01
Nigeria	2917	1.85	1.55	2.2	5666	1.49	1.31	1.70
Rwanda	999	1.52	1.11	2.08	1844	1.78	1.38	2.31
Senegal	1082	2.00	1.48	2.72	2123	1.55	1.22	1.98
Sierra Leone	445	1.61	0.96	2.68	945	1.39	0.96	2.01
Swaziland	280	2.11	0.76	5.90	568	1.44	0.74	2.80
Tanzania	647	1.27	0.80	2.01	1420	1.38	0.95	2.00
Uganda	820	1.65	1.16	2.34	1516	1.64	1.23	2.17
Zambia	442	2.33	1.42	3.81	883	1.72	1.18	2.52
Zimbabwe	315	1.04	0.51	2.14	706	1.88	0.88	3.99
**Asia**								
Bangladesh	666	1.32	0.90	1.93	1406	1.44	1.11	1.87
Cambodia	1238	2.28	1.59	3.28	2338	1.80	1.36	2.39
India	7800	1.97	1.74	2.23	14325	1.76	1.60	1.93
Indonesia	2210	1.30	0.92	1.82	3846	1.45	1.06	1.97
Maldives	939	2.26	1.49	3.44	1444	1.66	1.19	2.32

Nepal	821	1.56	1.08	2.25	1587	1.54	1.20	1.98
Pakistan	1866	1.66	1.27	2.16	2658	1.41	1.13	1.75
Philippines	1103	2.64	1.58	4.42	1816	1.76	1.16	2.66
**Americas**								
Bolivia	1360	2.09	1.54	2.84	2348	2.03	1.58	2.61
Colombia	2499	2.19	1.39	3.46	4161	1.70	1.21	2.38
Dominican Republic	2259	2.04	1.25	3.33	3421	1.26	0.81	1.95
Haiti	867	1.88	1.13	3.12	1537	1.72	1.23	2.41
Honduras	1672	2.42	1.71	3.44	2790	2.08	1.55	2.8
**Other**								
Albania	319	1.76	0.51	6.07	691	1.07	0.43	2.63
Armenia	383	0.98	0.43	2.25	590	0.92	0.44	1.92
Azerbaijan	655	1.34	0.65	2.78	981	1.33	0.71	2.5
Egypt	163	3.91	2.77	5.51	78	3.01	2.29	3.96
Jordan	1969	1.99	1.06	3.73	3070	1.33	0.74	2.37
Moldova	152	1.04	0.35	3.12	297	0.69	0.28	1.67

Kozuki N and Walker N. Exploring the association between short/long preceding birth intervals and child mortality: using reference birth interval children of the same mother as comparison.

Supplemental Table 2b: Sample sizes and odds ratios relative risk for long birth intervals, with neonatal mortality as outcome (reference birth interval: 24-<60 months)

	≥60 months				≥72 months			
country	Number of mothers contributing to analysis	OR	95% CI: Lower	95% CI: Upper	Number of mothers contributing to analysis	OR	95% CI: Lower	95% CI: Upper
**Africa**								
Benin	845	0.80	0.49	1.31	423	0.71	0.36	1.40
Burkina Faso	783	0.52	0.23	1.14	388	0.41	0.12	1.38
Cameroon	510	0.62	0.26	1.45	296	0.74	0.27	2.02
Chad	304	0.34	0.11	1.11	161	0.42	0.07	2.53
Congo-Brazzaville	545	0.56	0.20	1.53	338	0.93	0.25	3.44
DRC	568	0.64	0.24	1.70	321	1.56	0.45	5.46
Ethiopia	874	0.48	0.25	0.92	483	0.50	0.19	1.32
Ghana	433	0.97	0.41	2.28	263	1.56	0.47	5.16
Guinea	649	0.27	0.11	0.66	365	0.42	0.14	1.25
Guyana	359	0.89	0.24	3.24	231	0.40	0.06	2.48
Kenya	531	2.45	0.97	6.18	296	4.52	1.28	16.01
Lesotho	510	3.04	1.24	7.48	303	3.74	1.12	12.47
Liberia	677	0.52	0.22	1.23	432	0.38	0.12	1.23
Madagascar	1079	0.81	0.36	1.81	648	0.68	0.26	1.77
Malawi	1592	0.72	0.42	1.24	876	1.10	0.56	2.15
Mali	797	0.60	0.29	1.25	429	0.39	0.10	1.58
Mozambique	781	0.52	0.17	1.57	459	0.52	0.11	2.50
Namibia	739	4.55	1.76	11.79	442	13.49	2.45	74.36
Niger	620	2.02	0.80	5.14	319	1.91	0.45	8.11
Nigeria	2090	0.65	0.44	0.95	1213	0.92	0.57	1.46
Rwanda	445	0.51	0.21	1.25	215	0.33	0.09	1.17
Senegal	849	0.85	0.33	2.18	489	1.13	0.28	4.53
Sierra Leone	599	0.29	0.13	0.66	389	0.15	0.05	0.50
Swaziland	393	0.19	0.02	1.51	244	0.25	0.01	4.47
Tanzania	720	0.77	0.35	1.72	400	0.88	0.34	2.32
Uganda	475	0.52	0.25	1.09	262	0.74	0.27	2.04
Zambia	426	0.56	0.22	1.45	222	0.57	0.15	2.07
Zimbabwe	672	1.24	0.47	3.26	385	1.90	0.58	6.20
**Asia**								
Bangladesh	1053	0.66	0.42	1.04	641	0.50	0.25	1.01
Cambodia	1049	0.85	0.46	1.60	594	1.03	0.48	2.22
India	4415	0.48	0.36	0.64	2777	0.52	0.35	.75
Indonesia	3304	1.08	0.67	1.74	2149	0.89	0.48	1.66
Maldives	792	0.93	0.45	1.94	486	1.17	0.47	2.93

Nepal	461	0.60	0.23	1.56	214	0.65	0.19	2.28
Pakistan	604	0.83	0.41	1.71	305	0.54	0.21	1.43
Philippines	638	1.97	0.90	4.31	351	2.45	0.77	7.77
**Americas**								
Bolivia	1058	0.61	0.27	1.34	661	0.60	0.20	1.79
Colombia	2932	1.24	0.73	2.11	1899	1.35	0.67	2.71
Dominican Republic	1388	0.67	0.32	1.41	854	0.70	0.29	1.71
Haiti	450	0.25	0.08	0.79	262	0.21	0.05	0.86
Honduras	1045	1.32	0.55	3.20	591	3.36	1.00	11.30
**Other**								
Albania	319	0.39	0.06	2.69	691	---	---	---
Armenia	383	10.51	1.63	67.73	590	---	---	---
Azerbaijan	655	2.86	0.47	17.59	981	3.29	0.29	36.98
Egypt	1969	1.29	0.65	2.55	3070	0.95	0.32	2.83
Jordan	672	0.88	0.20	3.81	311	2.63	0.42	16.60
Moldova	249	8.48	0.68	105.17	144	2.71	0.09	82.42

Kozuki N and Walker N. Exploring the association between short/long preceding birth intervals and child mortality: using reference birth interval children of the same mother as comparison.

Supplemental Table 3: Mean and median percentage of women who have high completed fertility, defined as 5 or more live births (when limiting to women who were age 35 or over at time of DHS interview)

	Africa	Asia	Americas	North Africa/Central Asia/Europe
Mean	80.28%	59.71%	55.58%	30.97%
Median	85.95%	58.88%	67.94%	17.61%

Kozuki N and Walker N. Exploring the association between short/long preceding birth intervals and child mortality: using reference birth interval children of the same mother as comparison.

Supplemental Table 4: Summary of country-level average time of birth, by birth interval category

Birth interval	<18 months	<24 months	24-<60 months	≥60 months	≥72 months
Difference in average calendar year that the births in each birth interval category occurred, averaged across all datasets*	1.23	1.13	ref	2.62	3.05
Smallest country-level difference (yrs) in average calendar year that the births in each birth interval category occurred**	0.04	0.1	ref	1.31	1.55
Largest country-level difference (yrs) in average calendar year that the births in each birth interval category occurred***	3.11	2.61	ref	4.57	5.53

*First, the average calendar year of birth was calculated for each birth interval category. Second, the difference in average calendar year was calculated between the exposure birth interval categories and the reference birth interval category. Third, that difference was then averaged across all 47 datasets to produce the number in the table.

**This row reports data from the country that reported the smallest difference in the aforementioned second step.

**This row reports data from the country that reported the largest difference in the aforementioned second step.

Supplemental Table 5: Summary of country-level average birth order, by birth interval category

Birth interval	<18 months	<24 months	24-<60 months	≥60 months	≥72 months
Difference in average birth order, averaged across all datasets*	0.32	0.22	ref	0.43	0.53
Smallest country-level difference in average birth order**	0	0.02	ref	0.01	0.03
Largest country-level difference in average birth order***	0.9	0.52	ref	0.79	0.96

*First, the average birth order was calculated for each birth interval category. Second, the difference in average birth order was calculated between the exposure birth interval categories and the reference birth interval category. Third, that difference was then averaged across all 47 datasets to produce the number in the table.

**This row reports data from the country that reported the smallest difference in the aforementioned second step.

**This row reports data from the country that reported the largest difference in the aforementioned second step.
